# Abrupt warming and salinification of intermediate waters interplays with decline of deep convection in the Northwestern Mediterranean Sea

**DOI:** 10.1038/s41598-020-77859-5

**Published:** 2020-12-01

**Authors:** Félix Margirier, Pierre Testor, Emma Heslop, Katia Mallil, Anthony Bosse, Loïc Houpert, Laurent Mortier, Marie-Noëlle Bouin, Laurent Coppola, Fabrizio D’Ortenzio, Xavier Durrieu de Madron, Baptiste Mourre, Louis Prieur, Patrick Raimbault, Vincent Taillandier

**Affiliations:** 1 CNRS-Sorbonne Universités (UPMC Univ. Pierre et Marie Curie, Paris 06)-CNRS-IRD-MNHN, UMR 7159, Laboratoire d’Océanographie et de Climatologie (LOCEAN), Institut Pierre Simon Laplace (IPSL), 4 place Jussieu, 75005 Paris, France; 2 IOC UNESCO, Paris, France; 3 ENSSMAL, EcosysMarL, 16320 Alger, Algeria; 4 Aix-Marseille Université, Université de Toulon, CNRS, IRD, MIO UM 110, 13288 Marseille, France; 5 National Oceanography Centre, Southampton, UK; 6 ENSTA-Paristech, Laboratoire d’OcéSouthamptonanographie et de Climatologie (LOCEAN), Palaiseau, France; 7 CNRM, Universite de Toulouse, Meteo-France, CNRS, Toulouse, France; 8 Univ. Brest, CNRS, IRD, Ifremer, Laboratoire d’Oceanographie Physique et Spatiale (LOPS), IUEM, 29840 Brest, France; 9 CNRS-Sorbonne Universités (UPMC Univ. Pierre et Marie Curie, Paris 06), UMR 7093, Laboratoire d’Océanographie de Villefranche (LOV), Observatoire Océanologique de Villefranche/mer, Paris, France; 10 CNRS-Université de Perpignan Via Domitia, Centre de Formation et de Recherche sur les Environnements Méditerranéens (CEFREM), Perpignan, France; 11 SOCIB, Balearic Islands Coastal Observing and Forecasting System, Palma de Mallorca, Spain

**Keywords:** Climate sciences, Ocean sciences

## Abstract

The Mediterranean Sea is a hotspot for climate change, and recent studies have reported its intense warming and salinification. In this study, we use an outstanding dataset relying mostly on glider endurance lines but also on other platforms to track these trends in the northwestern Mediterranean where deep convection occurs. Thanks to a high spatial coverage and a high temporal resolution over the period 2007–2017, we observed the warming (+0.06 $^\circ$C year$^{-1}$) and salinification (+0.012 year$^{-1}$) of Levantine Intermediate Water (LIW) in the Ligurian Sea. These rates are similar to those reported closer to its formation area in the Eastern Mediterranean Sea. Further downstream, in the Gulf of Lion, the intermediate heat and salt content were exported to the deep layers from 2009 to 2013 thanks to deep convection processes. In 2014, a LIW step of +0.3 $^\circ$C and +0.08 in salinity could be observed concomitant with a weak winter convection. Warmer and more saline LIW subsequently accumulated in the northwestern basin in the absence of intense deep convective winters until 2018. Deep stratification below the LIW thus increased, which, together with the air–sea heat fluxes intensity, constrained the depth of convection. A key prognostic indicator of the intensity of deep convective events appears to be the convection depth of the previous year.

## Introduction

The Mediterranean Sea is a semi-enclosed sea characterized by a basin-scale cyclonic circulation, and by ubiquitous and energetic meso- and submesosale dynamics^1–8^. It is also one of the rare hotspots for deep water formation in the global ocean^9^, with several active sites of intermediate and deep water formation^10–15^. The time scales associated with its density-driven circulation are about 10 times shorter than those of the global ocean^16^. Besides, the Mediterranean Sea’s rapid and amplified responses to weather-related stress make it a hotspot when it comes to climate change and a key region for observing its impacts^17–21^.

The Mediterranean Sea exchanges heat and salt with the Atlantic Ocean through the Strait of Gibraltar. Fresh Atlantic Water (AW) in the surface layer flows in, while saltier and colder (on average over a year) water from the Mediterranean Sea flows out at depth. AW follows a cyclonic circulation along the northern continental slope of the western Mediterranean Sea and enters the Eastern basin through the Sicily Channel^3^. During this journey, it undergoes evaporation and mixing with resident waters, very likely due to vigorous mesoscale activity and vertical mixing during winter. AW then gradually becomes more saline^22–26^. In the eastern basin, AW is transformed into intermediate and deep waters that are relatively colder and saltier. This alteration is due to air–sea exchanges resulting from significant heat loss and evaporation in winter, as well as mixing with the surrounding Mediterranean waters. The deep water formation processes play an active role in ventilating the deep waters^27^ and—as in the global ocean—exporting carbon to the deep layers^28,29^.

Deep water formation also occurs in the northwestern Mediterranean Sea where it is driven by both intense winter atmospheric events (Mistral and Tramontane winds) and oceanic preconditioning. In the center of the northwestern Mediterranean basin, isopycnals are closer to the surface and favour deep mixing. This is associated to the basin-scale cyclonic circulation dominating this sub-basin^9,10^. This natural preconditioning also has a chaotic component due to the mesoscale variability^4,30,31^. The relatively fresh (and cold in winter) AW can mix with the underlying warm and salty Levantine Intermediate Water (LIW) to produce new Western Mediterranean Deep Water (WMDW) in the Gulf of Lion. LIW properties are thus critical for the deep ventilation process^32^. Vertical mixing has been reported to reach the seafloor at 2000–2500 m in some years^14^.

Formed in the Levantine basin in the eastern Mediterranean by similar processes to those forming deep waters^33^, the LIW follows a cyclonic path, under the surface AW, to reach the western Mediterranean^3,16^. The LIW then flows through the Sicily Channel and into the Tyrrhenian basin, reaching the northwestern Mediterranean about a decade after its formation^34^ after flowing along the western coasts of Corsica and Sardinia and to a smaller extent through the Corsica Channel. There has been evidence that LIW is becoming saltier and warmer in the eastern basin^35^ and in the Sicily Channel^36^. It is likely to have an impact on deep convection as it propagates towards the Gulf of Lion. This increase has also been reported in the Balearic Sea^37,38^, downstream of the LIW path through the deep convection area.

Intensified monitoring in the western Mediterranean highlighted an abrupt shift in LIW properties. Both the French “MOOSE” and the Spanish “Canales-SOCIB” glider endurance lines^39^ observed this shift. The data from these glider missions were combined with additional in-situ data sets available in the region (from ship Conductivity Temperature Depth profiles (CTDs), moorings, profiling floats, and eXpandable BathyThermographs (XBTs) to provide 10 years of multi-platform observations scattered throughout the Northwestern Mediterranean region. This data set was used to study the causes and consequences of the sudden increases in temperature and salinity detected in the Gulf of Lion in 2014. The signal was found to further propagate following the main current towards the Balearic Islands. Using such a multi-platform approach was efficient in characterizing this abrupt change first observed by the glider’s endurance lines because of the spatio-temporal coverage that was achieved. The combination of vertical ocean profiles data sets with mooring measurements from the deep convection zone has given a better understanding of the effects of deep convection events on intermediate layers at basin scale. This led to a reflection on the deep and bottom-reaching convection events that could occur in the future in the context of the global and Mediterranean changes.

## Results

**Figure 1:**
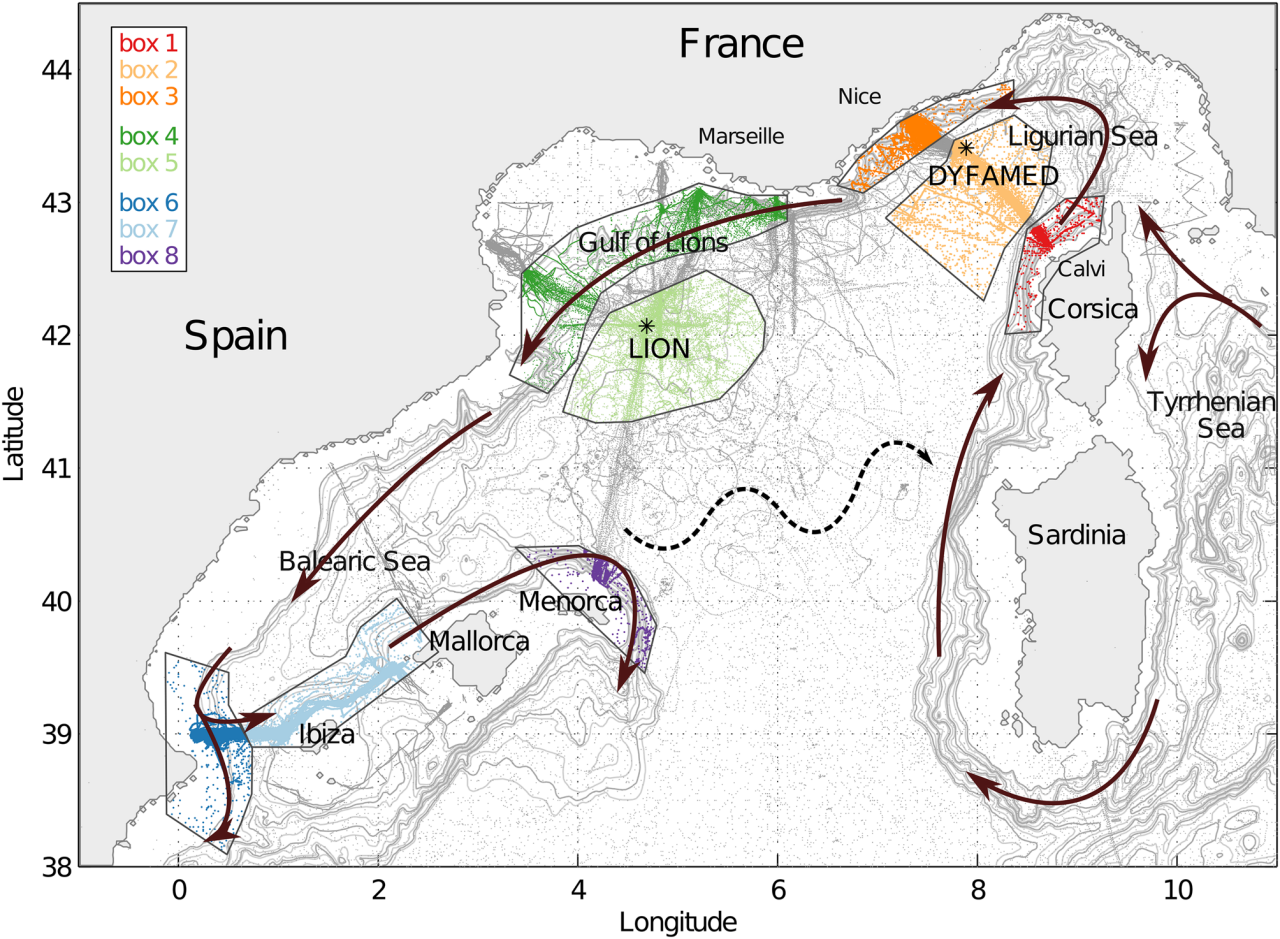
Color-coded regional areas: orange Ligurian Sea, green Gulf of Lion and blue Balearic Sea. Each region is decomposed in sub-regions defined by the general circulation. The scattered dots represent all the profiles collected between 01/01/2007 and 01/01/2018 in the respective regional areas in color and outside in grey. The two mooring locations (LION and DYFAMED) are indicated by the black stars. The LIW general circulation is indicated by the arrows and the north Balearic Front by the dashed one. The background map was generated with ETOPO5 (Data Announcement 88-MGG-02, Digital relief of the Surface of the Earth. NOAA, National Geophysical Data Center, Boulder, Colorado, 1988).

Thanks to this new set of high spatial and temporal resolution data in the northwestern Mediterranean, we now have a better understanding of the warming and salinification trends. The data were classified into regional areas (Fig. 1) throughout the cyclonic circulation in the northwestern Mediterranean Sea. Temperature and salinity time series of the LIW characteristics were computed for each region. The LIW flows at intermediate depths ($\sim$200–500 m) and is characterized by a subsurface salinity (and temperature) maximum.

### The Ligurian Sea

The LIW enters the Ligurian Sea before flowing within the Northern Current along the southern French coast towards the Balearic Sea (Fig. 1).

**Figure 2:**
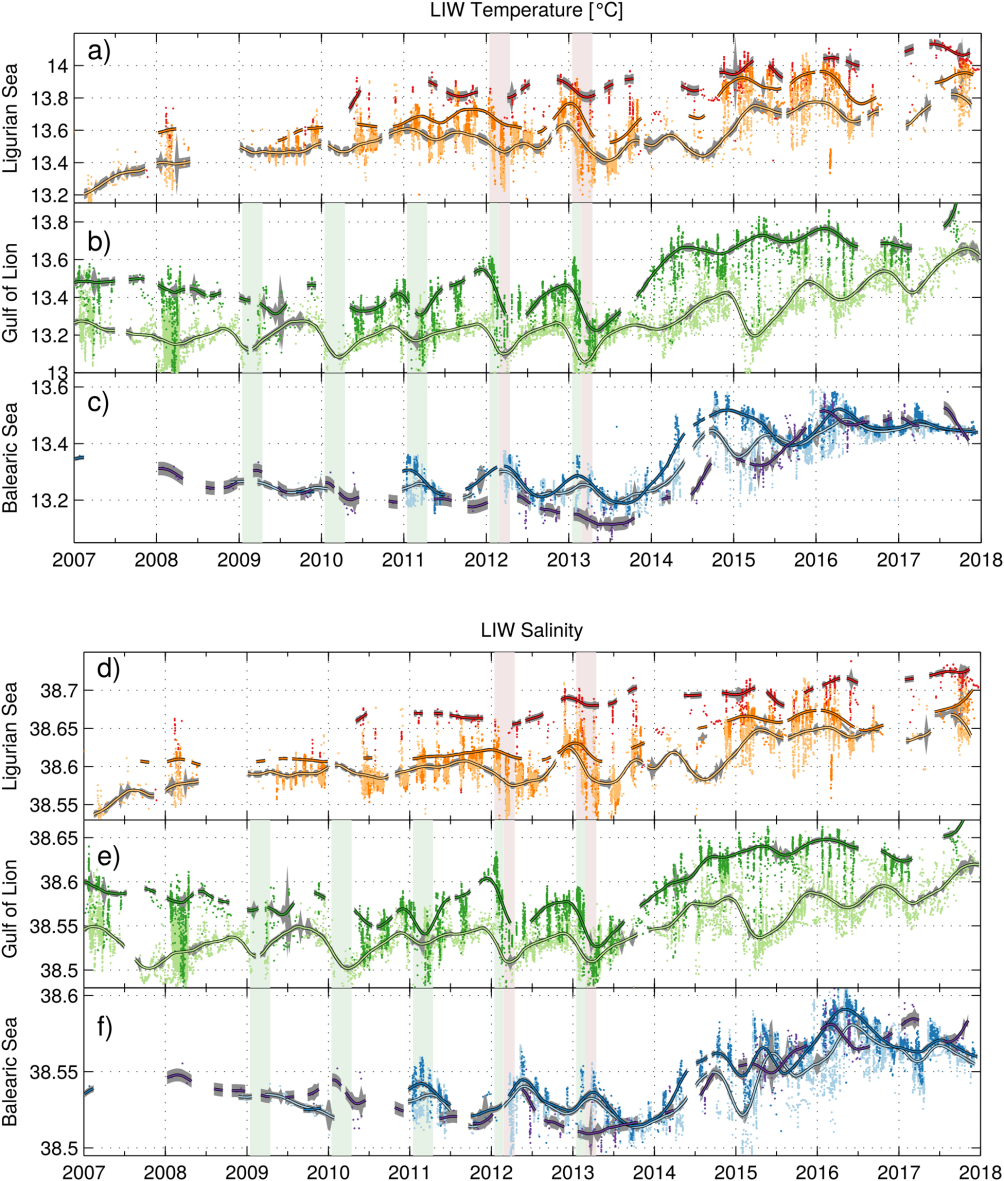
Temperature and salinity 2007–2018 time series in the: (**a**,**d**) Ligurian Sea, (**b**,**e**) Gulf of Lion and (**c**,**f**) Balearic Sea . The color-code is that of Fig. 1. The winters with deep convection in the Ligurian Sea and the Gulf of Lion are indicated by purple and green vertical patches, respectively. The black contoured curves represent the 90-day running mean and the grey patches around them the running standard error in each regional subset of data (the very large number of points explains its low value).

The high-resolution monitoring of the Ligurian Sea allows for a geographical classification of the LIW circulation associated with the Northern Current into three regions: two boundary regions, Area 1 ’Calvi’ and Area 2 ’Nice’, and one open ocean region: Area 3 ’offshore Ligurian’. From 2007 to 2017, the LIW temperature and salinity in these different areas consistently increased by $+0.06 \pm 0.01$ $^\circ$C year$^{-1}$ and $+0.012 \pm 0.02$ year$^{-1}$ (Fig. 2). These trends are consistent with the increase in heat and salt from the eastern basin reported by Schroeder et al.^36^ (+0.06$~^\circ$C year$^{-1}$ and +0.014 year$^{-1}$) in the Sicily Channel.

**Figure 3:**
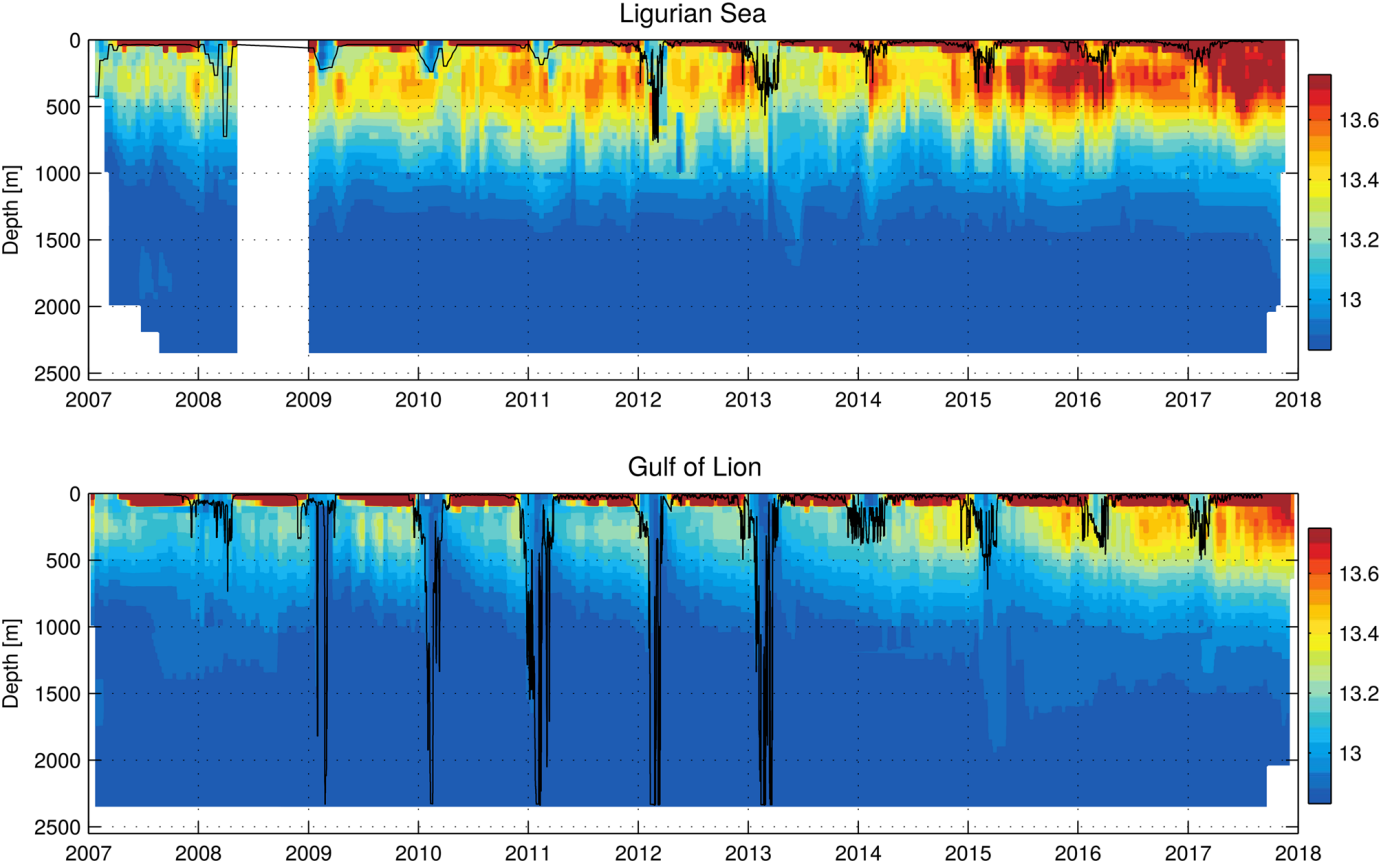
Depth-time diagrams of temperature at (top) DYFAMED (43.41$^\circ$ N 7.89$^\circ$ E) and (bottom) LION (42.04$^\circ$ N 4.68$^\circ$ E) for the Ligurian Sea and Gulf of Lion respectively. Here, all the profiles within a 15 km radius from the mooring location were used, and merged with the mooring line measurements. The black contour represents the mixed layer depth, computed as in Houpert et al.^14^.

Area 1 ’Calvi’ showed little variability for LIW around this linear trend, with values typical of the LIW entering the northwestern Mediterranean Sea. Area 2 ’Nice’ followed about the same pattern but the LIW is slightly less warm and less saline, as a result of mixing with resident and offshore waters following its path along the continental slope. The cross-correlation analysis of the detrended time series led to a circulation time from Area 1 ’Calvi’ to Area 3 ’Nice’ of about 40 days (equivalent to 7 cm s$^{-1}$, $R^2=0.92$) which is consistent with estimates of ocean currents at that depth^23^. The ’offshore Ligurian’ Area 2 exhibited similar trends but was more impacted by the convective events, notably in 2012 and 2013 (Fig. 2). The offshore LIW core properties were eroded during winter (Fig. 3), through mixing with the relatively fresh and cold overlaying AW. This effect was not long-lasting, as LIW advected from the Tyrrhenian Sea reconquered the intermediate layers during the following months (Figs. 2 and 3 ). A drop in the offshore Ligurian LIW temperature and salinity could be observed in summer 2014, possibly due to variations in the inflowing waters that showed a variability of the same order. The contrasts between the offshore and coastal areas remained relatively constant in the absence of convective events, likely due to the mesoscale activity leading to cross-shelf exchanges.

### Deep convection in the Gulf of Lion

LIW signals propagated from Area 3 ’Nice’ to Area 4 ’Gulf of Lion slope’ in 20 days (equivalent to 14 cm    s$^{-1}$, $R^2=0.78$) and were advected offshore into a region of potential deep convection. Acting as a sink for the heat and salt contained by LIW, deep convection events exported its properties to the shallow surface AW layer and more importantly in the deep layers, implying the warming and salinification of the WMDW^14,15,40,41^. This was the case during the period 2009–2013 (Fig. 2), when bottom-reaching convection occurred (Table 1). In winter, the LIW was mixed with the relatively fresher and colder overlaying AW, as well as with the relatively fresher and colder WMDW underneath. Subsequently, the LIW temperature and salinity increased from April to December, in both the offshore and shelf slope regions. The LIW characteristics thus continued to increase until the following convection episode that brought them back to low levels similar to the ones reached the previous winter. Hence, the winter mixing helps maintaining the local LIW properties the northwestern Mediterranean Sea.

A jump of the LIW temperature ($+0.30 \pm 0.04$ $^\circ$C) and salinity ($+0.08 \pm 0.01$) was observed in 2014. That year, the winter mixing did not reach great depths (i.e. the mixed layer reached a maximum depth of 430 m observed at the LION mooring location), limiting the export of heat and salt to the deep waters. Over the 2014–2017 period, the winter convection remained moderate and thus only slightly modified the LIW properties offshore every winter. Due to this reduced vertical mixing, temperature and salinity steadily increased both in the Gulf of Lion slope and offshore regions at similar rates as farther upstream (see the input observed in Fig. 2 in the red Area 1 ’Calvi’ time series), discrepancies likely resulting from the ubiquitous mesoscale activity.

**Figure 4:**
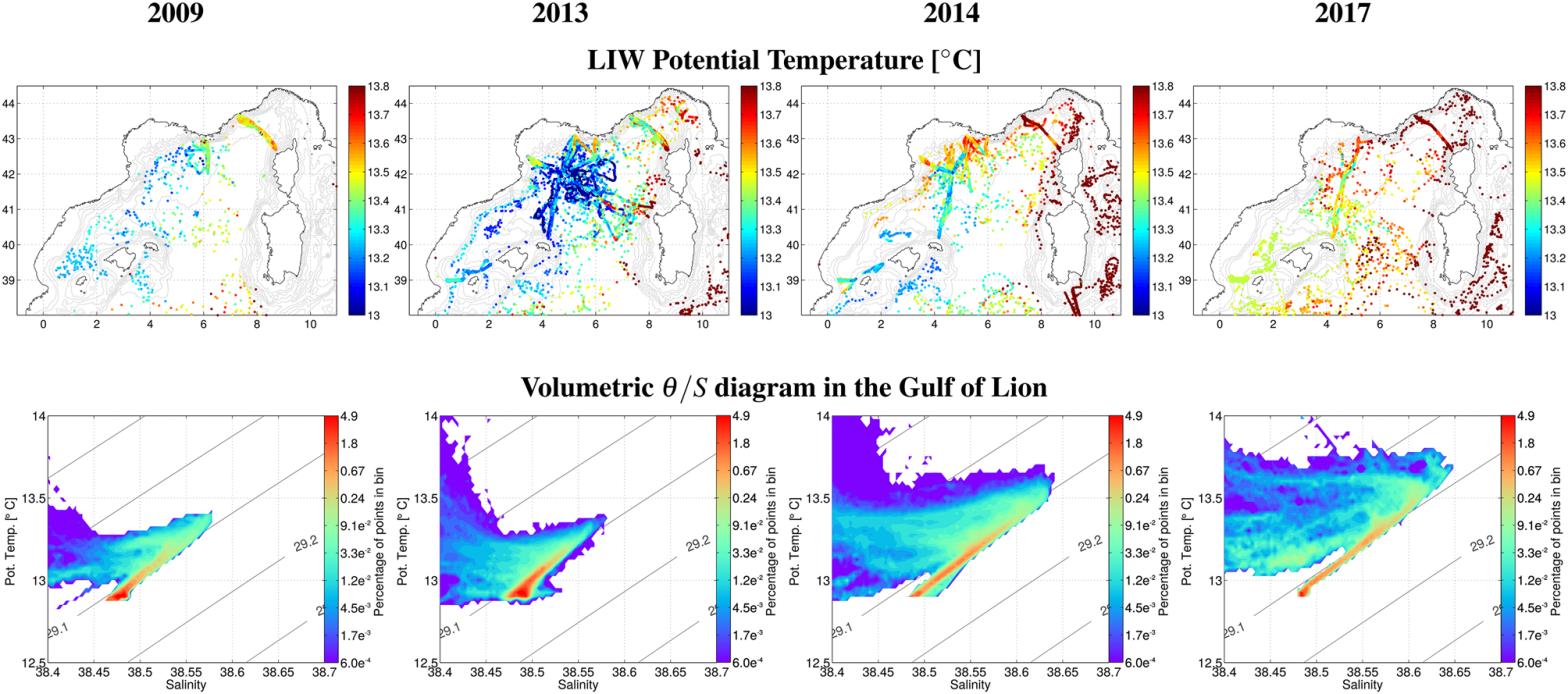
(top row) LIW core temperature in the northwestern Mediterranean Sea during the two contrasted periods (2009–2013 and 2014–2017). LIW temperature and salinity (not shown) remain constant prior to 2014, the first winter with no deep convection. The heat and salt then increased throughout the basin. (bottom row) Year by year volumetric $\theta -S$ diagrams with all profiles in Area 5 ’offshore Gulf of Lion’ (Fig. 1). Red accounts for a high density of points and thus volume of water. The period with deep convection (of these only 2009 and 2013 are shown) presents a high volume of waters with the same properties (WMDW), while in the following years the LIW occupies a larger portion of the water column, as the waters are distributed on the mixing line between the WMDW and LIW. The background map was generated with ETOPO5 (Data Announcement 88-MGG-02, Digital relief of the Surface of the Earth. NOAA, National Geophysical Data Center, Boulder, Colorado, 1988).

**Figure 5:**
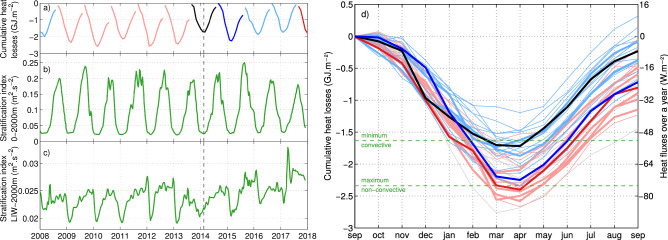
(**a**) Cumulative monthly heat losses to the atmosphere over each year at the LION mooring location, starting in September of the previous year and finishing in August (light red for convective years, light blue for non-convective years, the year labels on the x-axis are positioned on January of each particular year). The 2014 and 2015 (starting in September 2013 and 2014) non-convective years are indicated in black and dark blue, and the 2018 (starting in September 2017) return of convection in dark red (only the first part of winter 2018 is shown here, see (**d**) for the whole coverage). The stratification index (see Methods): (**b**) 0–2000 m and (**c**) LIW-2000 m at the LION mooring line location are also represented. The dashed black vertical line represents the time of the regime shift. (**d**) Cumulative monthly heat losses to the atmosphere over each year (starting in September of the previous year, see (**a**)), in light red for deep convective years (identified as year with convection reaching more than 1000 m deep), light blue for non-convective ones (when convection reached less than 1000 m deep) and in grey for unreported years, starting in 1979. The black line indicates the 2014 transition winter (cumulative monthly heat losses over a year starting in September 2013 with a minimum of − 1.71 GJ m$^{-2}$: equivalent to − 94.2 W m$^{-2}$ over 7 months after which that minimum is observed), the dark blue the 2015 ensuing year (− 2.24 GJ m$^{-2}$: equivalent to − 123.4 W m$^{-2}$ over 7 months). The years 2008–2018 are plotted in thicker lines than the 1979–2007 historical period. The return of convection in winter 2018 is indicated in dark red (cumulative minimum of − 2.39 GJ m$^{-2}$: equivalent to − 131.7 W m$^{-2}$ over 7 months). The minimum cumulative loss initiating deep convection was − 1.63 GJ m$^{-2}$: equivalent to − 104.8 W m$^{-2}$ over 6 months, the maximum with no deep convection was − 2.33 GJ m$^{-2}$: equivalent to − 128.4 W m$^{-2}$ over 7 months.

The 4 years without intense deep convection following 2013 allowed the LIW entering in the basin to preserve its core with high temperature and salinity, and to “invade” the intermediate layers in the Gulf of Lion (Figs. 3 and 4 ). The intermediate layers shifted rapidly to a warmer and more saline state. During the transition winter 2014, the cumulative monthly heat losses to the atmosphere over the year revealed a minimum compared to other years (Fig. 5a), which could explain the absence of deep mixing. In winter 2013, the stratification at the beginning of winter was similar (Fig. 5b), but the larger air–sea fluxes triggered an intense deep convective event^15^. The ensuing absence of intense deep convection events induced a slightly increasing stratification in the deep layers (Stratification Index $SI = \int _{0}^{2000} \frac{g}{\rho _0} \frac{\partial \rho }{\partial z} z dz$^42^, Fig. 5b,c) and particularly intense convective events would be needed to break that increased stability.

### Propagation to the Balearic Sea

The signals observed in 2014 in the Ligurian Sea and the Gulf of Lion then propagated to the Balearic Sea. The time lag of 3–5 months is consistent with recent observations^37,38^. The cross-correlation of the detrended time series provided an estimate of 80 days (equivalent to 9.4 cm s$^{-1}$, $R^2=0.87$) for the anomalies to propagate from Area 4 ’Northern Current slope’ to Area 6 ’Ibiza Channel’ (Fig. 2). The signal then propagated to Area 7 ’Ibiza-Mallorca’ lagged by an additional 10–20 days (equivalent to 5.7 cm s$^{-1}$, $R^2=0.81$), and to Area 8 ’Menorca’ region after 40 more days (equivalent to 8.6 cm s$^{-1}$, $R^2=0.84$). At the end of the year 2014, temperature and salinity drops were observed in Area 7 ’Ibiza-Mallorca’ (Fig. 2). This could be due to the formation of a relatively large amount of Western Intermediate Water (WIW) during winter, mixing with the underlying LIW and eroding its characteristics. Following on, between 2014 and 2017, rapid warming and salinification of the intermediate layers of the Balearic Sea took place, but slightly slower than in the other regions. This could be explained by the greater presence of WIW^37^, but small inputs of older, and thus less warm and saline, LIW from the south^38^ could also play a role. This signal then propagated south toward the Algerian Basin (Fig. 4).

## Discussion

From 2009 to 2013, bottom-reaching convection occurred every year^14^. The intermediate waters were ventilated regularly and remained relatively stable in temperature ($\sim 13.2$ $^\circ$C) and salinity ($\sim 38.52$) in the Gulf of Lion and downstream (Fig. 4). Upstream, in the Ligurian basin and the Sicily Channel^43^, the LIW had become warmer and more saline similarly to its source in the eastern Mediterranean Sea^35^. Between 2014 and 2017, in the absence of intense deep convection in the Gulf of Lion, the intermediate waters became warmer and more saline throughout the basin (Fig. 4). The heat and salt content at intermediate levels leapt to a new state. LIW also became more deficient in oxygen^44^. As the LIW continued to invade a larger portion of the water column due to moderate winter mixing limited to 400–700 m depths, the water volume distribution between the deep and intermediate waters also changed. The surface phytoplankton dynamics was also impacted in the absence of intense ventilation^45,46^, as the upward flux of nutrients was reduced. A similar regime shift occurred in the Ligurian Sea, where there was no convection below the LIW between 1990 and 2004. This lack of deep mixing induced an accumulation of heat and salt in the middle of the basin^47^, before deep convection occurred in 2005.

Deep convection is controlled by the oceanic preconditioning and the intensity of heat loss to the atmosphere^9^. Changing the preconditioning thus has an impact on deep convective events. In order to assess the preconditioning, we will hereafter refer to years starting in September of the previous year when the ocean starts losing heat to the atmosphere. The weaker heat losses of winter 2014 (cumulative monthly heat losses over a year starting in September 2013 with a minimum of − 1.71 GJ m$^{-2}$—equivalent to − 94.2 W m$^{-2}$ over the 7 months after which that minimum is observed—compared to − 2.42 GJ m$^{-2}$—equivalent to − 133.3 W m$^{-2}$ over 7 months—in 2013 and − 2.55 GJ m$^{-2}$—equivalent to − 163.9 W m$^{-2}$ over 6 months—in 2012) played a major role in transitioning the basin to a new state (Fig. 5a). The stratification index below the LIW core (characterized by a temperature or salinity maximum between 300 and 350 m depth) and 2000 m depth increased (Fig. 5c), though the stratification index between 0 and2000 m depth has changed little (Fig. 5b). This increase likely led to the absence of convection deeper than 1000 m in 2015 despite strong fluxes (cumulative monthly heat losses over a year with a minimum of − 2.24 GJ m$^{-2}$—equivalent to − 123.4 W m$^{-2}$ over 7 months—). Consequently, stronger atmospheric forcing would have been required to trigger convection down to the seafloor, concurring with the predictions of a weakened Mediterranean overturning circulation^19^.

To assess the impact of the intensity of heat losses in deep convection, historical heat losses were confronted with historical Mixed Layer Depths (MLD)^48^, starting in 1979. The year with the lowest cumulative monthly heat losses that could trigger convection deeper than 1000 m had a minimum of − 1.63 GJ m$^{-2}$—equivalent to − 104.8 W m$^{-2}$ over 6 months—; while the year with the highest cumulative monthly heat losses that could not be associated to a deep convective winter had a minimum of − 2.33 GJ m$^{-2}$—equivalent to − 128.4 W m$^{-2}$ over 7 months—. This overlap attests of the key role of the ocean preconditioning.

**Figure 6:**
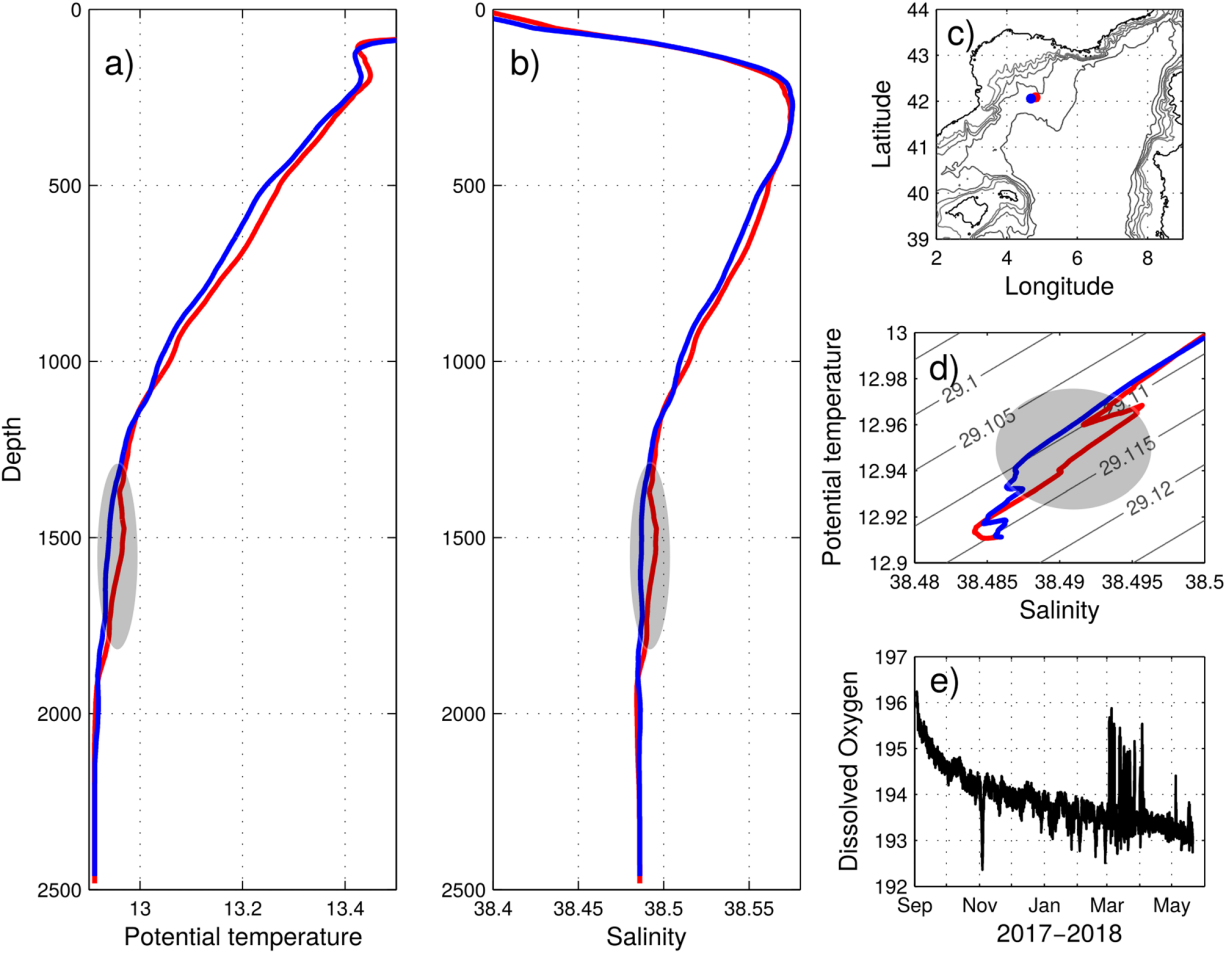
(**a**) Potential temperature and (**b**) salinity depth profiles collected during the MOOSE-GE 2018 cruise in spring: (**c**) leg 1, stations 040 in blue (21/04/2018 19h15 UTC) and 041 in red (21/04/2018 21h52 UTC). (**d**) $\theta -S$ diagram for the two corresponding profiles. The newly formed deep waters detected on profile 041 in red are marked by the grey patch. They were detected down to 1800 m. (**e**) Dissolved oxygen ($\upmu$ mol kg$^{-1}$) time series at 2000 m between 04/09/2017 and 22/05/2018 recorded at the LION mooring line.

We hypothesize a key prognostic indicator of the intensity of a deep convective event is the convection depth of the previous year. As the intermediate heat and salt contents increase, sufficiently high air–sea fluxes would be needed for the mixing to penetrate below the LIW layer and trigger a new deep convection event. A similar event occurred in the northern Atlantic after a 4-year shutdown in the Labrador Sea^49^. This seems to have happened in winter 2018 to some extent (Fig. 5d) when strong heat losses (cumulative monthly net heat losses over a year with a minimum of − 2.39 GJ m$^{-2}$—equivalent to − 131.7 W m$^{-2}$ over 7 months—) triggered convection down to at least 1800 m depth (see the collected profiles the ensuing spring after some restratification in Fig. 6). The mixing initiated deep water ventilation as indicated by the brief increase in the oxygen concentration at 2000 m depth detected at the LION mooring location. To estimate the convection area extension, we use the satellite image showing the largest area with low chlorophyll concentration and apply a 0.15 mg m$^{-3}$ contour (based on Herrmann et al.^50^). Globcolor and ESA-CCI-OC-v4.2 8-day products respectively gave maximum surface areas of 13,400 km$^2$ and 10,400 km$^2$, about half of the surface reported by Houpert et al.^14^ or Testor et al.^15^ for strong convective years. In 2018, the convective episode seemed to have been limited in space and time as only a small volume of newly ventilated waters was detected (notably by the MOOSE network^51^), likely due to the increased deep stratification and despite very intense heat losses.

Our analysis shows that the spatio-temporal coverage of temperature and salinity profiles in the northwestern Mediterranean Sea allows an almost continuous regional description of the basin evolution on a 10-days basis between 2007 and 2017. This was mainly due to a shift in ocean observing capabilities allowed by sustained glider operations, repeated deployments of profiling floats and the maintenance of several mooring lines, in the deep convection area in particular. It demonstrates the potential of an integrated and multi-platform approach, and the continuation of such sustained efforts of observation during the next decades is crucial as it will allow us to distinguish and/or confirm any long-term trend in temperature and salinity in the ocean. It will also enable the monitoring of deep convection and its potential shutdown. A number of studies have addressed different aspects of the western Mediterranean Transient^52,53^, either focusing on a restricted area, temporal interval or process. This unique set of observations collected during a decade (2007–2017) contributes to the understanding of the temporal and spatial evolution of thermohaline variability in the western Mediterranean Sea. By modifying the thermohaline circulation of the Mediterranean Sea, these changes will definielty impact the pelagic and abyssal ecosystems structure as well as the carbon cycle. The correct simulation of this climate shift is a significant challenge to the climate modelling community and a prerequisite for accurate projections of the ocean state in the next decades. This first documentation of heterogeneous distribution of LIW in the basin, both along the boundary circulation and in the open-sea, thereby provides a new benchmark for the improvement and validation of ocean models.

Climate projections do not agree on the occurrence of deep convection in future climate scenarios, but most tend to point towards a decrease^19,20,54,55^. However, they all predict an increase in the heat and salt content of the Mediterranean basin^21,56,57^. The intermediate waters of the western basin are yearly regulated by the occurrence and intensity of deep convective events, which seem to have maintained the western Mediterranean LIW characteristics in a quasi-steady state for several years prior to 2014. Time series of temperatures measured in the Planier and Lacaze-Duthiers canyons (i.e. within the LIW along the continental slope of the Gulf of Lion) even suggest that such an increase had not occurred since observations began in 1993. The LIW is becoming warmer and saltier at its formation point in the eastern Mediterranean^35^, so it could be expected this observed increases will continue at least at the same rate in upcoming years if no intense deep convection events regulate that. Also, as the LIW temperature and salinity properties increase, so do the properties of the WIW^37,58^ and of the WMDW formed by their mixing. Our study shows a crucial role of the LIW in the ventilation of the deep waters in the western Mediterranean Sea that highlights important remote forcing and constraints on a local important process.

Furthermore, this quick and intense warming could likely have an important impact on the biogeochemical cycles and on the pelagic marine life^59,60^ as well as on the benthic ecosystems^61^. The increasing heat and salt contents of the intermediate and deep water masses of the western Mediterranean basin^58,62,63^ could in turn alter the characteristics of the Mediterranean outflow into the Atlantic Ocean. Dedicated studies will be required to understand the impact of these changes on the ocean circulation at a larger scale.

## Methods

Some 166 083 vertical profiles collected by gliders, Argo profiling floats, CTDs and XBTs in the northwestern Mediterranean Sea over the 2007–2017 period in the eight different regions of interest were used in this study (colored points in Fig. 1). The profiles were harmonized using the method described in Bosse et al.^7^ and Testor et al.^15^ to generate an unified database. This method ensures errors in temperature and salinity to be smaller than 0.01 $^\circ$C and 0.01, respectively.

A regional approach was adopted to assess both the variability and the evolution of water masses along their course. The focus was set on the eight regions presented in Fig. 1 along the cyclonic circulation of the northwestern Mediterranean Sea, comprising offshore and alongshore components. In these regional areas, the impact of climate change and the effect of deep convection on the evolution of the LIW was assessed. LIW core properties were extracted from each profile and plotted in the time series presented in Fig. 2. The core of the LIW was considered to be either the salinity and temperature maxima deeper than the 29 kg m$^{-3}$ isopycnal. This method allowed for a better tracking of the LIW directly in T/S space rather than extracting the properties at a fixed depth. In winter, if the water column is fully homogeneous after being mixed, there is no LIW in a strict sense, and the temperature and salinity of the homogeneous water column were then used to define the LIW characteristics.

Merging the French and Spanish efforts resulted in a very high spatial and temporal resolution dataset, mainly enabled by the sustained deployments of gliders. Analysing the intermediate waters regime shift by only retrieving one point per profile was thus possible. The computation of the running mean time series with a very large number of points resulted in a very low standard deviation, despite the discrepancies in temporal coverage. It also enabled the detrended cross-correlation of the different time series to compute propagation times.

The DYFAMED (43.41 N 7.89 E) and LION (42.04 N 4.68 E) deep mooring lines (from $\sim$200 m down to the seafloor) were complemented in the surface layers as in Houpert et al.^14^ with the AZUR (43.38 N–7.83 E) and LION (42.06 N–4.64 E) Météo France surface buoys equipped by sub-surface instruments (0–150 m), allowing for a thorough vertical coverage of the water column. The mixed layer depth was computed as in Houpert et al.^14^, applying a first criterion of $\delta \theta =0.1$ $^\circ$C with a reference level of 10 m for the first 300 m. If the MLD was deeper than 300 m, a second criterion of $\delta \theta =0.01$ $^\circ$C with a reference level of 300 m was used. Historical MLDs prior 2007 were retrieved from Somot et al.^48^.

The interpolation presented in Fig. 3 relied on a two-layer analysis : objective analyses were performed for the surface (0–700 m) and deep (700 m-bottom) layers separately, using 20 days/50 m and 3 months/200 m scales, respectively. These scales were chosen due to the different variabilities of the two layers of the water column. All profiles within a 15 km radius of the LION mooring line (approximately equivalent to the deformation radius in the northwestern Mediterranean) were used as well as the mooring line data, to perform these objective analyses with the most comprehensive data set. Heat fluxes were retrieved from ERA-Interim monthly means of daily accumulated heat fluxes re-analyses^64^. Globcolor and ESA-CCI-OC-v4.2 8-day products were respectively retrieved on http://hermes.acri.fr/ and https://esa-oceancolour-cci.org/version-42-data-release.

We used the Stratification Index (SI) computed in several Mediterranean studies^1,48,65–67^ and derived from the Turner equation^42^. The SI at depth *h* was computed as $SI = \int _{0}^{h} \frac{g}{\rho _0} \frac{\partial \rho }{\partial z} z dz$ and indicates the ability of mixing the water column. A large SI attests of a stratified water column, a negative SI indicates an unstable water column about to overturn and a *SI* of zero at depth *h* a perfectly mixed water column down to said depth. It is similar to the Convection Resistance (CR) used in the north Atlantic^68,69^ which suggests the amount of buoyancy to be removed for the mixed layer to reach depth *h*: $CR = \int _{-h}^{0} \sigma dz - h\sigma$. It is a partial integration of the stratification index modulo a $g/\rho _0$ factor and represents the same quantity.

**Table 1:** Maximum depth reached by winter convection between 2008 and 2017 and corresponding time, at the LION mooring site using the MLD criterion from Houpert et al.^14^.

	2008	2009	2010	2011	2012	2013	2014	2015	2016	2017
Date of maximum depth	07/04	24/02	16/02	08/02	23/02	23/02	09/12	06/03	20/03	12/02
Maximum convection depth	730	2330	2330	2330	2330	2330	420	710	460	500

## References

[1] Lascaratos A, Nittis K (1998). A high resolution 3-d numerical study of intermediate water formation in the levantine sea. J. Geophys. Res..

[2] Nadia P, Masetti E (2000). Variability of the large scale general circulation of the mediterranean sea from observations and modelling: A review. Palaeogeogr. Palaeoclimatol. Palaeoecol..

[3] Millot C, Taupier-Letage I (2005). Circulation in the Mediterranean Sea. Hanb. Environ. Chem..

[4] Testor, P. & Gascard, J.-C. Post-convection spreading phase in the northwestern mediterranean sea. *Deep Sea Res. Part I Oceanogr. Res. Papers* **869–893**. 10.1016/j.dsr.2006.02.004 (2006).

[5] Menna M, Poulain P-M (2010). Mediterranean intermediate circulation estimated from argo data in 2003–2010. Ocean Sci..

[6] Malanotte-Rizzoli P (2014). Physical forcing and physical/biochemical variability of the mediterranean sea: a review of unresolved issues and directions for future research. Ocean Sci..

[7] Bosse A (2016). Scales and dynamics of submesoscale coherent vortices formed by deep convection in the northwestern mediterranean sea. J. Geophys. Res. Oceans.

[8] Mauri E (2019). On the variability of the circulation and water mass properties in the eastern levantine sea between september 2016-august 2017. Water.

[9] Marshall J, Schott F (1999). Open-ocean convection: observations, theory, and models. Rev. Geophys..

[10] MEDOC-Group. Observation of formation of deep water in the mediterranean sea, 1969. *Nature* **225**, 1037–1040 (1970). 10.1038/2271037a0.

[11] Lascaratos A, Roether W, Nittis K, Klein B (1999). Recent changes in deep water formation and spreading in the eastern mediterranean sea: a review. Prog. Oceanogr..

[12] Manca B, Ibello V, Pacciaroni M, Scarazzato P, Giorgetti A (2006). Ventilation of deep waters in the adriatic and ionian seas following changes in thermohaline circulation of the eastern mediterranean. Clim. Res..

[13] Özsoy E (2014). Deep-water variability and inter-basin interactions in the eastern mediterranean sea. Geophys. Monogr. Ser..

[14] Houpert L (2016). Observations of open-ocean deep convection in the northwestern mediterranean sea: Seasonal and interannual variability of mixing and deep water masses for the 2007–2013 period. J. Geophys. Res. Oceans.

[15] Testor P (2018). Multiscale observations of deep convection in the northwestern mediterranean sea during winter 2012–2013 using multiple platforms. J. Geophys. Res. Oceans.

[16] Durrieu de Madron, X. *et al.* Marine ecosystems’ responses to climatic and anthropogenic forcings in the Mediterranean. *Prog. Oceanogr.* **91**, 97–166 (2011). 10.1016/j.pocean.2011.02.003.

[17] Bethoux J, Gentili B (1999). Functioning of the mediterranean sea: past and present changes related to freshwater input and climate changes. J. Mar. Syst..

[18] Cacho I, Grimalt J, Canals M (2002). Response of the western mediterranean sea to rapid climatic variability during the last 50,000 years: A molecular biomarker approach. J. Mar. Syst..

[19] Somot, S., Sevault, F. & Déqué, M. Transient climate change scenario simulation of the mediterranean sea for the twenty-first century using a high-resolution ocean circulation model. *Clim. Dyn.* **27** (2006). 10.1007/s00382-006-0167-z.

[20] Somot, S., Sevault, F., Déqué, M. & Crépon, M. 21st century climate change scenario for the mediterranean using a coupled atmosphere-ocean regional climate model. *Global Planet. Change* **63**. 10.1016/j.gloplacha.2007.10.003 (2008).

[21] Giorgi F, Lionello P (2008). Climate change projections for the mediterranean region. Global Planet. Change.

[22] Font, J., Millot, C., Salas Pérez, J. D. J., Julià, A. & Chic, O. The drift of modified atlantic water from the alboran sea to the eastern mediterranean. *Sci. Mar.* **62**, 211–216 (1998). 10.3989/scimar.1998.62n3211.

[23] Millot C (1999). Circulation in the western mediterranean sea. J. Mar. Syst..

[24] Testor P (2005). The mean circulation of the southwestern Mediterranean Sea: Algerian Gyres. J. Geophys. Res. Oceans.

[25] Schroeder, K. *et al.* Circulation of the mediterranean sea and its variability. *Clim. Mediterranean Region Past Future* **187–256**. 10.1016/B978-0-12-416042-2.00003-3 (2012).

[26] Pisano, A. *et al.* New evidence of mediterranean climate change and variability from sea surface temperature observations. *Remote Sens.* **12**. 10.3390/rs12010132 (2020).

[27] Coppola, L. *et al.* Observation of oxygen ventilation into deep waters through targeted deployment of multiple argo-o 2 floats in the north-western mediterranean sea in 2013. *J. Geophys. Res. Oceans* **122**. 10.1002/2016JC012594 (2017).

[28] Fröb F (2016). Irminger sea deep convection injects oxygen and anthropogenic carbon to the ocean interior. Nat. Commun..

[29] Touratier, F. *et al.* Role of deep convection on anthropogenic co2 sequestration in the gulf of lions (northwestern mediterranean sea). *Deep Sea Res. Part I: Oceanogr. Res. Papers* **113**. 10.1016/j.dsr.2016.04.003 (2016).

[30] Bosse, A. *et al.* Spreading of levantine intermediate waters by submesoscale coherent vortices in the northwestern mediterranean sea as observed with gliders. *J. Geophys. Res. Oceans* **120** (2015). 10.1002/2014JC010263.

[31] Waldman, R., Somot, S., Herrmann, M., Sevault, F. & Erik Isachsen, P. On the chaotic variability of deep convection in the mediterranean sea. *Geophys. Res. Lett.* **45** (2018). 10.1002/2017GL076319.

[32] Grignon, L., Smeed, D., L. Bryden, H. & Schroeder, K. Importance of the variability of hydrographic preconditioning for deep convection in the gulf of lion, nw mediterranean. *Ocean Sci. Disc.* **7**, 51–90 (2010). 10.5194/os-6-573-2010.

[33] Lascaratos A, Williams R, Tragou E (1993). A mixed-layer study of the formation of levantine intermediate water. J. Geophys. Res..

[34] Millot C (2005). Circulation in the mediterranean sea: Evidences, debates and unanswered questions. Sci. Mar..

[35] Ozer, T., Gertman, I., Kress, N., Silverman, J. & Herut, B. Interannual thermohaline (1979–2014) and nutrient (2002–2014) dynamics in the levantine surface and intermediate water masses, se mediterranean sea. *Global Planet. Change*. 10.1016/j.gloplacha.2016.04.001 (2016).

[36] Schroeder, K. *et al.* Rapid response to climate change in a marginal sea. *Sci. Rep.* **7**. 10.1038/s41598-017-04455-5 (2017). 10.1038/s41598-017-04455-5 PMC5481423 28642495

[37] Juza, M. *et al.* Characterization of changes in western intermediate water properties enabled by an innovative geometry-based detection approach. *J. Mar. Syst.* **191**. 10.1016/j.jmarsys.2018.11.003 (2018).

[38] Barceló-Llull, B. *et al.* Temporal and spatial hydrodynamic variability in the mallorca channel (western mediterranean sea) from eight years of underwater glider data. *J. Geophys. Res. Oceans*. 10.1029/2018JC014636 (2019).

[39] Testor, P. *et al.* Oceangliders: A component of the integrated global ocean observing system. *Front. Mar. Sci.* **6**. 10.3389/fmars.2019.00422 (2019).

[40] Bethoux J, Gentili B, Raunet J, Tailliez D (1990). Warming trend in the western mediterranean deep water. Nature.

[41] Rohling EJ, Bryden HL (1992). Man-induced salinity and temperature increases in western mediterranean deep water. J. Geophys. Res..

[42] Turner, J. S. Buoyancy effects in fluids. *Cambridge University Press*. 10.1002/2016JC012671 (1973).

[43] Schroeder, K., Chiggiato, J., L. Bryden, H., Borghini, M. & Ben Ismail, S. Abrupt climate shift in the western mediterranean sea. *Sci. Rep.* **6**, 23009 (2016). 10.1038/srep23009. 10.1038/srep23009 PMC4786855 26965790

[44] Coppola, L. *et al.* Seasonal and inter-annual variationsof dissolved oxygen in the northwestern mediterranean sea (dyfamed site). *Prog. Oceanogr.* **162**, 1. 10.1016/j.pocean.2018.03.001 (2018).

[45] Kessouri, F. *et al.* Vertical mixing effects on phytoplankton dynamics and organic carbon export in the western mediterranean sea. *J. Geophys. Res.*. 10.1002/2016JC012669 (2018).

[46] Mayot, N. *et al.* Influence of the phytoplankton community structure on the spring and annual primary production in the northwestern mediterranean sea. *J. Geophys. Res. Oceans* **122**. 10.1002/2016JC012668 (2017).

[47] Prieur, L., D’Ortenzio, F., Taillandier, V. & Testor, P. Physical oceanography of the ligurian sea. in: Migon C., Sciandra, A. & Nival, P. (eds.), the mediterranean sea in the era of global change (volume 1),- evidence from 30 years of multidisciplinary study of the ligurian sea. *ISTE Science Publishing LTD,* 49–78 (2020). 10.1002/9781119706960.ch3.

[48] Somot, S. *et al.* Characterizing, modelling and understanding the climate variability of the deep water formation in the north-western mediterranean sea. *Clim. Dyn.*. 10.1007/s00382-016-3295-0 (2016).

[49] Gelderloos R, Straneo F, Katsman C (2012). Mechanisms behind the temporary shutdown of deep convection in the labrador sea: lessons from the great salinity anomaly years 1968–1971. J. Clim..

[50] Herrmann, M., Auger, P.-A., Ulses, C. & Estournel, C. Long-term monitoring of ocean deep convection using multisensors altimetry and ocean color satellite data. *J. Geophys. Res. Oceans*. 10.1002/2016JC011833 (2017).

[51] Coppola, L., Raimbault, P., Mortier, L. & Testor, P. Monitoring the environment in the northwestern mediterranean sea. *Eos* **100**. 10.1029/2019EO125951 (2019).

[52] Schroeder, K. *et al.* An extensive western mediterranean deep water renewal between 2004 and 2006. *Geophys. Res. Lett.* **35**. 10.1029/2008GL035146 (2008).

[53] Schneider, A., Tanhua, T., Roether, W. & Steinfeldt, R. Changes in ventilation of the mediterranean sea during the past 25 year. *Ocean Sci. (OS)* **10**. 10.5194/os-10-1-2014 (2014).

[54] Brodeau, L. & Koenigk, T. Extinction of the northern oceanic deep convection in an ensemble of climate model simulations of the 20th and 21st centuries. *Clim. Dyn.* **46**. 10.1007/s00382-015-2736-5 (2016).

[55] Macías, D., Garcia-Gorriz, E. & Stips, A. Deep winter convection and phytoplankton dynamics in the nw mediterranean sea under present climate and future (horizon 2030) scenarios. *Sci. Rep.* **8**. 10.1038/s41598-018-24965-0 (2018). 10.1038/s41598-018-24965-0 PMC5919909 29700363

[56] Planton, S. *et al.* The climate of the mediterranean region in future climate projections. *Clim. Medit. Region* **449–502**. 10.1016/B978-0-12-416042-2.00008-2 (2012).

[57] Somot, S., Jorda, G., Harzallah, A. & Darmaraki, S. Sub-chapter 1.2.3. the mediterranean region under climate change: a scientific update. *IRD Editions* (2016). 10.4000/books.irdeditions.23100.

[58] Vargas-Yáñez M (2012). Extreme western intermediate water formation in winter 2010. J. Mar. Syst..

[59] Coma R (2009). Global warming-enhanced stratification and mass mortality events in the mediterranean. Proc. Nat. Acad. Sci. U.S.A..

[60] Tamburini C (2013). Deep-sea bioluminescence blooms after dense water formation at the ocean surface. PLoS ONE.

[61] Danovaro, R. Climate change impacts on the biota and on vulnerable habitats of the deep mediterranean sea. *Rendiconti Lincei. Scienze Fisiche e Naturali*10.1007/s12210-018-0725-4 (2018).

[62] Borghini M, Bryden H, Schroeder K, Sparnocchia S, Vetrano A (2014). The mediterranean is becoming saltier. Ocean Sci..

[63] Durrieu de Madron, X. *et al.* Deep sediment resuspension and thick nepheloid layer generation by open-ocean convection. *J. Geophys. Res. Oceans* **122** (2017). 10.1002/2016JC012062.

[64] Dee, D. *et al.* The era-interim reanalysis: Configuration and performance of the data assimilation system. *Q.J.R. Meteorol. Soc.* **137**, 553–597 (2011). 10.1002/qj.828.

[65] Herrmann MJ, Somot S, Sevault F, Estournel C, Déqué M (2008). Modeling the deep convection in the northwestern Mediterranean sea using an eddy-permitting and an eddy-resolving model: Case study of winter 1986–1987. J. Geophys. Res. Oceans.

[66] Herrmann, M., Beuvier, J., Sevault, F. & Somot, S. What induced the exceptional 2005 convection event in the northwestern mediterranean basin? answers from a modeling study. *J. Geophys. Res. C Oceans* **115**. 10.1029/2010JC006162 (2010).

[67] , Li, L., Sevault, F. & Somot, S. Interannual variability of deep convection in the northwestern mediterranean simulated with a coupled aorcm. *Clim. Dyn.* **41** (2012). 10.1007/s00382-012-1527-5.

[68] Bailey D, Rhines P, Häkkinen S (2005). Formation and pathways of north atlantic deep water in a coupled ice-ocean model of the arctic-north atlantic oceans. Clim. Dyn..

[69] Frajka-Williams E, Rhines P, Eriksen C (2014). Horizontal stratification during deep convection in the labrador sea. J. Phys. Oceanogr..

